# Disseminated paracoccidioidomycosis in a liver transplant patient^☆^^☆☆^

**DOI:** 10.1016/j.abd.2020.07.011

**Published:** 2021-03-15

**Authors:** Flávia de Oliveira Valentim, Giuliane Minami Tsutsui, Luciana Patrícia Fernandes Abbade, Silvio Alencar Marques

**Affiliations:** Faculty of Medicine, Universidade Estadual Paulista, Botucatu, SP, Brazil

**Keywords:** Dermatology, Liver, Paracoccidioidomycosis, Transplant

## Abstract

Paracoccidioidomycosis is an endemic systemic mycosis caused by *Paracoccidioides brasiliensis* complex and *P. lutzii*. It is a rare disease in non-HIV-induced immunosuppressed individuals. In organ transplant recipients, it is more frequently associated with immunosuppression after kidney transplantation. In a liver transplant patient, only one case has been published in the literature to date. The present report comprises the case of a 47-year-old female patient with disseminated skin lesions associated with signs and symptoms of systemic involvement of paracoccidioidomycosis that manifested one year after liver transplantation and under an immunosuppression regimen with tacrolimus and mycophenolate mofetil.

Paracoccidioidomycosis is a systemic mycosis caused by species of thermally-dimorphic fungi of the *Paracoccidioides brasiliensis* complex or by *P. lutzii*.^1^, ^2^ It clinically presents as the acute/subacute juvenile form, adult chronic form, and the one related to immunosuppression. The gold standard diagnosis is the detection of the fungus by direct mycological examination, histopathological analysis, or fungal isolation in culture.

In immunosuppressed individuals, it is more frequently associated with HIV infection, followed by solid, hematological neoplasms and, finally, post-solid organ transplants, particularly kidney transplantation under immunosuppression induced by prednisone, azathioprine, or cyclosporine. As seen in the researched literature to date (PubMed, SciELO, LILACS), there is only one described case after liver transplantation, and in this case, using tacrolimus.^3^, ^4^, ^5^, ^6^, ^7^

This is the report of a 47-year-old female patient, a former resident in the rural area of the state of São Paulo, who was submitted to a liver transplant one year before, due to cirrhosis of alcoholic etiology associated with hepatitis B, undergoing specific therapy consisting of 1 mg/day of tacrolimus and 720 mg/day of mycophenolate mofetil. She was referred to the Dermatology service for multiple lesions, with a 15-day exacerbation associated with high fever, malaise, and weakness. She also reported weight loss, lack of appetite, dry cough, and dyspnea for approximately three months. On physical examination, she had numerous acneiform and solid erythematous-normochromic papules (with an ulcerated center or covered with crusts) spread throughout the skin, including the face (Fig. 1).

**Figure 1:**
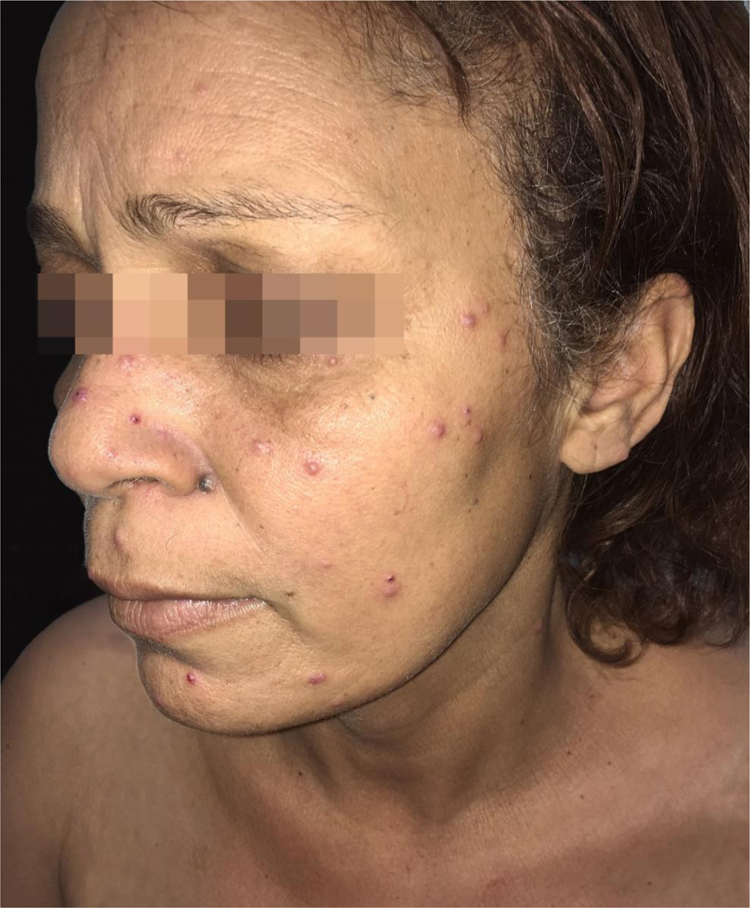
Paracoccidioidomycosis and liver transplantation. Acneiform lesions and erythematous papules, some with an ulcerated center, disseminated on the face.

The diagnostic hypotheses were histoplasmosis, cryptococcosis, and paracoccidioidomycosis. The histopathological analysis of a skin lesion biopsy showed epidermal hyperplasia and granulomatous and dermal suppurative inflammatory infiltrate, with the presence of fungal elements inside giant cells (Fig. 2). Grocott-Gomori staining showed multi-budding fungal cells, characteristic of the *Paracoccidioides* spp genus (Fig. 3).

**Figure 2:**
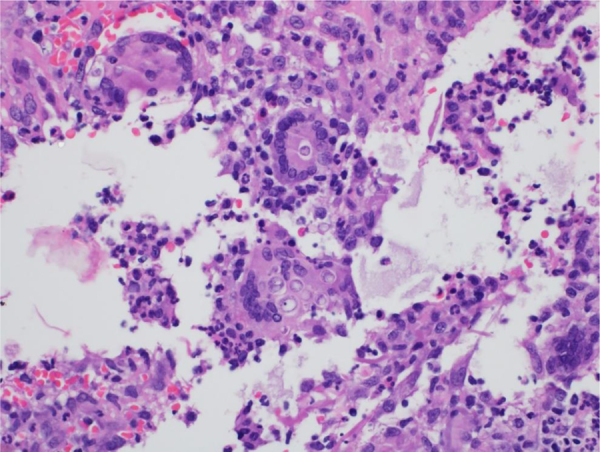
Paracoccidioidomycosis and liver transplant: inflammatory process detail, showing fungal cells in the giant cell cytoplasm (Hematoxylin & eosin, ×100).

**Figure 3:**
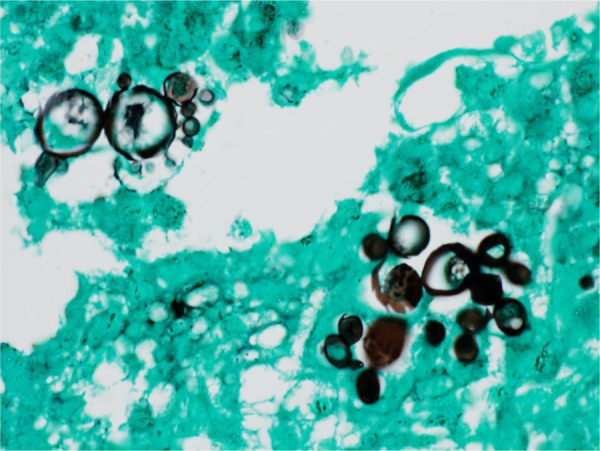
Paracoccidioidomycosis and liver transplantation: multi-budding fungal cells, characteristic of the *Paracoccidioides* genus (Grocott-Gomori. Immersion).

The biopsy fragment culture, general biochemical analyses, blood cultures, and serology, including anti-HIV, and anti-Paracoccidioides, were normal or negative. The chest CT (Fig. 4) showed coalescent pulmonary consolidations, septal thickening and excavated lesions in the lower portions and lymph node enlargement, compatible with the diagnosis of paracoccidioidomycosis due to the likely reactivation of a quiescent pulmonary focus.^8^

**Figure 4:**
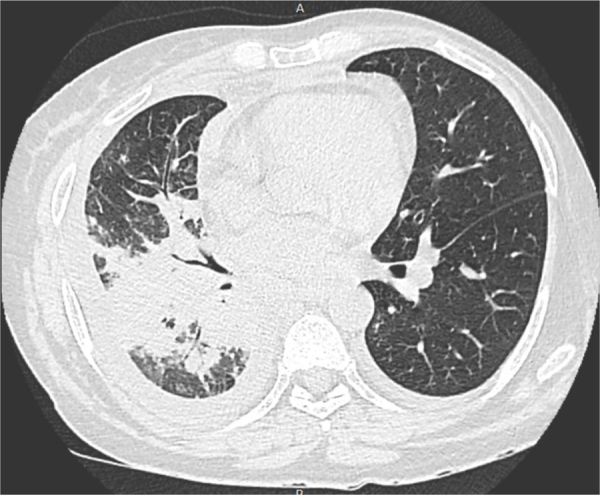
Paracoccidioidomycosis and liver transplantation: CT scan showing peribronchial and perivascular parenchymal consolidations.

Treatment was carried out with 3 mg/kg/day of liposomal amphotericin B for 4 weeks, on an in-hospital basis, requiring transfer to the ICU due to hemodynamic instability associated with the amphotericin B infusions and the precarious overall status. The patient was discharged after stabilization and clinical recovery, remission of paracoccidioidomycosis, and was prescribed itraconazole 200 m g/day as a maintenance treatment.

In immunosuppressed patients, the clinical picture of histoplasmosis, cryptococcosis, paracoccidioidomycosis and even disseminated sporotrichosis converges to similar manifestations: a relatively short history of fever, lack of appetite, weight loss, and asthenia, followed by or concomitant with the appearance of skin lesions showing an acneiform, disseminated ulcerated papular-nodular or ulcerated-necrotic pattern, frequent facial involvement, associated with signs and symptoms of systemic involvement.^3^, ^9^ The prognosis of paracoccidioidomycosis is reserved in post-transplant cases, resulting from the use and maintenance of immunosuppressants, disease dissemination and possible complications associated with therapy. The options of itraconazole, sulfamethoxazole-trimethoprim, amphotericin B deoxycholate, or liposomal are defined by the overall clinical picture and availability.^10^ The present report of paracoccidioidomycosis in a patient under immunosuppressive medication highlights treatment difficulties, but also the good final result.

## Financial support

None declared.

## Authors’ contributions

Flávia de Oliveira Valentim: Approval of the final version of the manuscript; conception and planning of the study; drafting and editing of the manuscript; intellectual participation in the propaedeutic and/or therapeutic conduct of the studied case; critical review of the literature.

Giuliane Minami Tsutsui: Approval of the final version of the manuscript; critical review of the manuscript.

Luciana Patrícia Fernandes Abbade: Approval of the final version of the manuscript; intellectual participation in the propaedeutic and/or therapeutic conduct of the studied case; critical review of the manuscript.

Silvio Alencar Marques: Approval of the final version of the manuscript; drafting and editing of the manuscript; intellectual participation in the propaedeutic and/or therapeutic conduct of the studied case; critical review of the literature; critical review of the manuscript.

## Conflicts of interest

None declared.
